# Tumor-promoting effects of pancreatic cancer cell exosomes on THP-1-derived macrophages

**DOI:** 10.1371/journal.pone.0206759

**Published:** 2018-11-01

**Authors:** Samuel S. Linton, Thomas Abraham, Jason Liao, Gary A. Clawson, Peter J. Butler, Todd Fox, Mark Kester, Gail L. Matters

**Affiliations:** 1 Department of Biochemistry and Molecular Biology, Pennsylvania State University College of Medicine, Hershey, Pennsylvania, United States of America; 2 Department of Neural and Behavioral Sciences, Pennsylvania State University College of Medicine, Hershey, Pennsylvania, United States of America; 3 Department of Public Health Sciences, Pennsylvania State University College of Medicine, Hershey, Pennsylvania, United States of America; 4 Department of Pathology, Pennsylvania State University College of Medicine, Hershey, Pennsylvania, United States of America; 5 Department of Engineering, Pennsylvania State University, University Park, Pennsylvania, United States of America; 6 Department of Pharmacology, University of Virginia, Charlottesville, Virginia, United States of America; University of South Alabama Mitchell Cancer Institute, UNITED STATES

## Abstract

Pancreatic ductal adenocarcinoma (PDAC) tumor growth is enhanced by tumor-associated macrophages (TAMs), yet the mechanisms by which tumor cells and TAMs communicate are not fully understood. Here we show that exosomes secreted by PDAC cell lines differed in their surface proteins, lipid composition, and efficiency of fusing with THP-1-derived macrophages *in vitro*. Exosomes from AsPC-1, an ascites-derived human PDAC cell line, were enriched in ICAM-1, which mediated their docking to macrophages through interactions with surface-exposed CD11c on macrophages. AsPC-1 exosomes also contained much higher levels of arachidonic acid (AA), and they fused at a higher rate with THP-1-derived macrophages than did exosomes from other PDAC cell lines or from an immortalized normal pancreatic ductal epithelial cell line (HPDE) H6c7. Phospholipase A_2_ enzymatic cleavage of arachidonic acid from AsPC-1 exosomes reduced fusion efficiency. PGE_2_ secretion was elevated in macrophages treated with AsPC-1 exosomes but not in macrophages treated with exosomes from other cell lines, suggesting a functional role for the AsPC-1 exosome-delivered arachidonic acid in macrophages. Non-polarized (M0) macrophages treated with AsPC-1 exosomes had increased levels of surface markers indicative of polarization to an immunosuppressive M2-like phenotype (CD14^hi^ CD163^hi^ CD206^hi^). Furthermore, macrophages treated with AsPC-1 exosomes had significantly increased secretion of pro-tumoral, bioactive molecules including VEGF, MCP-1, IL-6, IL-1β, MMP-9, and TNFα. Together, these results demonstrate that compared to exosomes from other primary tumor-derived PDAC cell lines, AsPC-1 exosomes alter THP-1-derived macrophage phenotype and function. AsPC-1 exosomes mediate communication between tumor cells and TAMs that contributes to tumor progression.

## Introduction

Exosomes are extracellular vesicles composed of a phospholipid bilayer that are secreted by most cells in the body [1]. Exosomes can interact with and deliver their macromolecular cargo, including proteins, DNA, mRNAs and microRNAs, to a variety of target cells and are a recognized vehicle of intercellular communication, both within local microenvironments as well as between tumors and distant tissues [2, 3]. Exosomes interact with cells through surface-exposed proteins which play a role in the selective uptake of exosomes by target cells [4] and the organotropism of metastatic cancer cells [5]. Pancreatic cancer-derived exosomes have been shown to prime the pre-metastatic niche in the liver through their actions on liver-resident macrophages [6]. Exosomes from melanoma, breast cancer, colon cancer, leukemia and glioma cells have been shown to have local pro-tumoral effects, and cancer cell exosomes can stimulate angiogenesis, reprogram stromal cells and promote tumor evasion of the adaptive immune system [7].

Many studies on pancreatic cancer exosomes have focused on circulating exosomes; evaluating their use as tumor biomarkers, characterizing protein, mRNA, or microRNA content, or assessing the pro-metastatic phenotypic and functional changes they induce in target cells at metastatic sites [6, 8]. Although phosphatidylserine-containing exosomes have been detected in the plasma of KIC and KPC mice [9], little is known about the lipid composition of human PDAC exosomes or if bioactive lipids from tumor exosomes can modulate the function of other tumor microenvironment (TME) cells to facilitate primary tumor growth and metastatic spread. Pancreatic cancer is characterized by a persistent, low-grade inflammatory TME and increased numbers of tumor-associated macrophages. In particular, the level of immunosuppressive (M2) TAMs in pancreatic tumors is inversely correlated with patient survival [10]. It is likely that these pro-tumoral TAMs receive multiple signals from other cells, including tumor cells, within the local TME [11].

In these studies, we isolated and characterized exosomes secreted by various PDAC cell lines cultured *in vitro*. The PDAC cell lines used in this study were AsPC-1, BxPC-3, PANC-1, and MIA PaCa-2 (MP-2) as well as the immortalized human pancreatic ductal epithelial (HPDE) cell line H6c7. These cell lines differ in their growth rate, invasiveness and metastatic potential [12, 13]. Recent studies demonstrated that compared with other PDAC cell lines, AsPC-1 cells are more metastatic to both liver and lungs after splenic injection [14]. We compared the ability of exosomes secreted by these various pancreatic cell lines to fuse with and modify the function of isolated macrophages. Specifically, we asked whether exosomal proteins are involved in docking exosomes with macrophages, whether exosomal lipids play a role in exosome-macrophage fusion and what effect exosome-macrophage fusion has on downstream macrophage functions that could affect the TME.

## Materials and methods

### Cultured cell lines

All cell lines were obtained from ATCC, grown at 37°C with 5% CO_2_, and STR verified yearly by ATCC. THP-1 cells (a human monocytic cell line) represent an established *in vitro* model commonly used to examine TAM-tumor cells interactions [15]. AsPC-1 cells were cultured in modified RPMI 1640 containing 10% FBS, 10 mM HEPES, 1 mM sodium pyruvate, and 13.9 mM D-glucose. Both THP-1 and BxPC-3 cells were cultured in 10% FBS-containing RMPI 1640, and PANC-1 and MIA-PaCa2 cell lines were cultured in 10% FBS-containing DMEM. The HPDE cell line H6c7, a gift from Dr. M.S. Tsao, University Health Network in Toronto, was maintained in keratinocyte serum-free medium (ThermoFisher Scientific) [16]. Each cell line was seeded into a 10-chamber CellSTACK factory (Corning Inc.), and at 80% confluence standard culture medium was replaced with serum-free medium. After 48 hours, spent cell culture medium (SCM) was collected and used for subsequent exosome purifications.

### Exosome isolation

To eliminate cellular debris that could contaminate downstream analysis of exosomal proteins, lipids, or secreted factors, sequential centrifugation was used to purify the secreted exosomes. SCM was centrifuged twice at 500 x *g* for 10 minutes at 4°C to pellet large cellular debris, and smaller debris was then pelleted at 10,000 x *g* for 30 minutes. The final supernatant was loaded into thinwall polypropylene ultracentrifuge tubes (10 mL/tube) (Beckman Coulter Inc.), underlayed with 20 mM Tris/30% sucrose in deuterium oxide (1 mL/tube), and centrifuged at 100,000 x *g* for 90 minutes at 4°C to pellet the exosomes. The tubes were pierced through the bottom with an 18-gauge needle and the sucrose layer was drawn into the syringe. The sucrose layers were pooled and diluted with excess 1X calcium- and magnesium-free phosphate buffered saline (PBS), and the exosomes were again pelleted at 100,000 x *g* for 90 minutes. The exosome pellet was resuspended in PBS and stored at -80°C. Exosome protein concentration was determined using a NanoOrange Protein Quantitation Kit (ThermoFisher Scientific), and total exosomal protein was used to normalize all other exosome comparisons.

### Exosome size analysis and visualization of exosomes by transmission electron microscopy (TEM)

Exosome size was measured using a Zetasizer Nano S (Malvern Instruments Ltd.). For TEM, 5 μL of exosome suspension was placed on a piece of parafilm and a formvar-coated copper grid was floated on the drop for 20 minutes at room temperature. The copper grid was blotted quickly on filter paper, placed on 4% paraformaldehyde in 0.1 M sodium phosphate buffer, pH 7.3, and washed by transferring to three separate PBS drops for one minute each. After placing in 1% glutaraldehyde in 0.1 M sodium phosphate buffer for 5 minutes, the grid was blotted quickly and moved to distilled water for 2 minutes. The grid was then washed four times with PBS and placed in 1% uranyl acetate for 20 seconds. Excess uranyl acetate was removed by blotting and the grid was imaged by transmission electron microscopy on a JEM-1400Plus (JEOL USA, Inc.).

### Immunoblot analysis of exosomal proteins

Equivalent amounts of total exosomal protein (30 μg) were resolved by SDS-PAGE and transferred to a polyvinylidine fluoride membrane. Primary antibodies used were: ICAM-1 (Cell Signaling Technology, #4915), flotillin-1 (D2V7J, Cell Signaling Technology, #18634), EpCAM (D1B3, Cell Signaling Technology, #2626), and CD9 (D8O1A, Cell Signaling Technology, #13174). Primary antibodies were diluted 1:1,000 in 5% BSA/TBST, and secondary HRP-conjugated antibodies were diluted 1:5,000 in 5% BSA/TBST. Target proteins were detected with an enhanced chemiluminescent substrate (ThermoFisher Scientific). The pan-exosomal marker flotillin-1 was used as a loading control.

### STtimulated emission depletion (STED) microscopy

THP-1 monocytes were differentiated into non-polarized (M0) macrophages with PMA (Cayman Chemical) [17]. After treating with 150 nM PMA-containing growth medium for 24 hours, PMA-containing medium was replaced with standard culture media and the THP-1 cells were allowed to recover for 24 hours. For co-localization studies, PMA-differentiated THP-1-derived macrophages were treated with 30 μg of AsPC-1 exosomes. After 4 minutes, cells were rinsed three times with PBS and fixed with ice-cold 100% methanol for 5 minutes. Following fixation, cells were washed three times with PBS for 5 minutes, blocked with 2% BSA in PBS and incubated with primary antibodies against CD11c (Invitrogen, #MA11C5, host: hamster) and ICAM-1 (Cell Signaling Technology, #4915T, host: rabbit) diluted 1:250 in 2% BSA/PBS at 4°C overnight. Cells were washed three times with PBS for 5 minutes and incubated for 1 hour with Alexa Fluor 568-conjugated goat anti-hamster secondary antibody (Invitrogen, #A-11011) and Alexa Fluor 532-conjugated goat anti-rabbit secondary antibody (Invitrogen, #A-11009) each diluted 1:250 in 2% BSA/PBS. After washing with PBS for 5 minutes, coverslips were mounted using ProLong Gold Antifade (Invitrogen, #P36930). Slides were kept at 4°C protected from light prior to imaging.

STED sub-diffraction microscopy was performed using Leica AOBS SP8 system integrated with 3XSTED module. STED images of fluorescently labeled cells were acquired using a STED 100x/ 1.40 oil immersion objective lens optimized for the overlay of excitation and STED laser beams. The laser lines used for excitation were 561 nm (for Alexa 568) and 514 nm (for Alexa 532), which were produced by 80 MHz pulsed white light laser (Leica AOBS SP8 module). The laser line used for depletion was continuous wave 660 nm. The respective emission signals were collected sequentially using AOBS tunable filters at 566–650 nm for Alexa 568 and 519–548 nm for Alexa 532. All images were generated using HyD detectors (with time gated option) in descanned mode. Backscattered emission signals from the sample were delivered through the AOBS tunable filter (to remove irradiated laser), the detection pinhole set to 0.73 Airy unit (to obtain optimal lateral and axial resolutions), spectral dispersion prism, and finally to the HyD detectors. The appropriate HyD gain level was then selected to obtain the pixel intensities within range of 0–255 (8-bit images) using a color gradient function. Images were acquired with pixel size satisfying the Nyquist sampling criteria and were line averaged 8 times with 3 times frame accumulation. Deconvolution of STED image datasets was performed using Huygens software (SVI) using theoretically generated point spread functions and the iterative deconvolution method based on the Classical Maximum Likelihood Algorithm (CMLE).

### Exosome lipidomic analysis

Beginning with equivalent amounts of exosomal protein (100 ug), glycerophospholipids were extracted using methyl-*tert*-butyl ether as described [18]. Briefly, extracted lipids were separated on a reversed-phase C8 column (Waters) and analyzed on a TripleTOF 5600 mass spectrometer (AB Sciex). The resulting lipid peak areas measured in extracted exosomal lipid samples were compared with those of the internal standards. After separation of glycerophospholipids, fatty acid analysis was also performed as described.

### Fusion assay

The rate of exosome fusion with THP-1-derived macrophages was measured by fluorescence dequenching utilizing the dimeric, self-quenching lipophilic dye octadecyl rhodamine B chloride (R18; Thermo Fisher Scientific) intercalated into the exosome membrane [19]. Fusion of R18-stained exosomes with THP-1 macrophage membranes separates the dye dimers and increases fluorescence.

To generate fluorescent exosomes, 10 μg of exosomes were incubated with 1 μL of 1 mM R18 in 1 mL of 1X MES fusion buffer (10 mM MES, pH 7.4, 124 mM NaCl, 5 mM KCl) for 30 minutes at 37°C. R18-stainined exosomes were separated from free R18 using Sephadex G-75 (GE Healthcare Bio-Science) and concentrated using a 10 kDa cut-off filter (EMD Millipore). Enzymatic removal of AA from exosomes was done by pre-treating with an equal volume of 0.03 ug/uL human phospholipase A2 (PLA_2_) (Origene) at 37°C for 30 minutes prior to staining with R18 and column purification.

To measure the rate at which exosomes fused with M0 macrophages, stained exosomes (untreated or PLA_2_ pre-treated) were added to a cuvette and placed in a SpectraMax M2 spectrophotometer (Molecular Devices) set at 37°C. Baseline fluorescence (560 nm excitation, 590 nm emission) was measured continuously for 15 minutes. THP-1-derived macrophages (7.5x10^5^ cells suspended in 500 μL 1X MES fusion buffer) were then spiked into the cuvette. The fusion reaction was stopped with 1 mL of 2X dequenching buffer (0.6% Triton X-100 and 120 nM octyl-beta-glucoside). Fusion was reported as % fluorescence dequenching = 100*((F–F_i_)/(F_max_-F_i_)), with F = fluorescence measured at a specific time point, F_i_ = the average fluorescence during the first 15 minutes, and F_max_ = the average fluorescence during the final 3 minutes following addition of an equal volume of 2X dequenching buffer.

### THP-1 macrophage polarization, staining and flow cytometry

To generate non-polarized (M0) macrophages, 4x10^5^ THP-1 monocytes were differentiated with PMA for 24 hours, after which the PMA-containing medium was replaced with 10% FBS-containing 1X RPMI growth medium and the cells were allowed to recover for 24 hours.

M0 macrophages were then treated with equivalent amounts of exosomes, based on 30 ug of exosomal protein. After 72 hours, cell culture medium was removed and stored at -80°C for analysis of secreted chemokines and cytokines (see below), and cells were detached using Accutase (Innovative Cell Technologies). After pre-treatment with Fc Block (BD Biosciences), 1x10^5^ cells were incubated for 30 minutes at 4°C with fluorochrome-conjugated primary antibodies against the following proteins: CD14 (ThermoFisher Scientific), HLA-DR (BD Biosciences), CD163 (R&D Systems), CD80 (Bio-Techne), and CD206 (BD Biosciences). Cells were washed twice with FACS buffer (0.8% BSA in PBS), resuspended in 0.5% PFA and stored at 4°C protected from light before being acquired on a LSRFortessa flow cytometer (BD Biosciences). Positive controls for M1 macrophage polarization were 20 ng/mL LPS and 20 ng/mL IFNɣ and for M2 macrophage polarization were 20 ng/mL IL-4 and 20 ng/mL IL-13. M1 and M2 polarization controls were treated for 24 hours. Data from at least 3 independent experiments were analyzed using FlowJo software (TreeStar).

### Immunoassay for secreted factors

Spent cell culture medium from exosome-treated THP-1-derived macrophages was used to assess the secretion of PGE_2_, VEGF, MCP-1, IL-6, IL-1β, MMP-9 and TNFα using enzyme-linked immunosorbent assays (Enzo Life Sciences for PGE_2_ and Meso Scale Diagnostics for VEGF, MCP-1, IL-6, IL-1β, MMP-9, and TNFα). For PGE_2_ cell culture supernatants were undiluted, and for VEGF, MCP-1, IL-6, IL-1β, MMP-9, and TNFα cell culture supernatants were diluted four-fold with buffer supplied by the manufacturer. Data from at least 3 independent experiments are represented as mean ± SEM.

### Statistical analyses

Data are presented as mean ± standard error of the mean or mean ± standard deviation as denoted in the figure legends. Comparison of the means between groups utilized unpaired two-tailed *t*-tests or one-way ANOVA with Prism 6.0 software (GraphPad), and *p*<0.05 was considered significant.

## Results

### Characterization of exosomes secreted by pancreatic cancer cell lines

**Fig 1:**
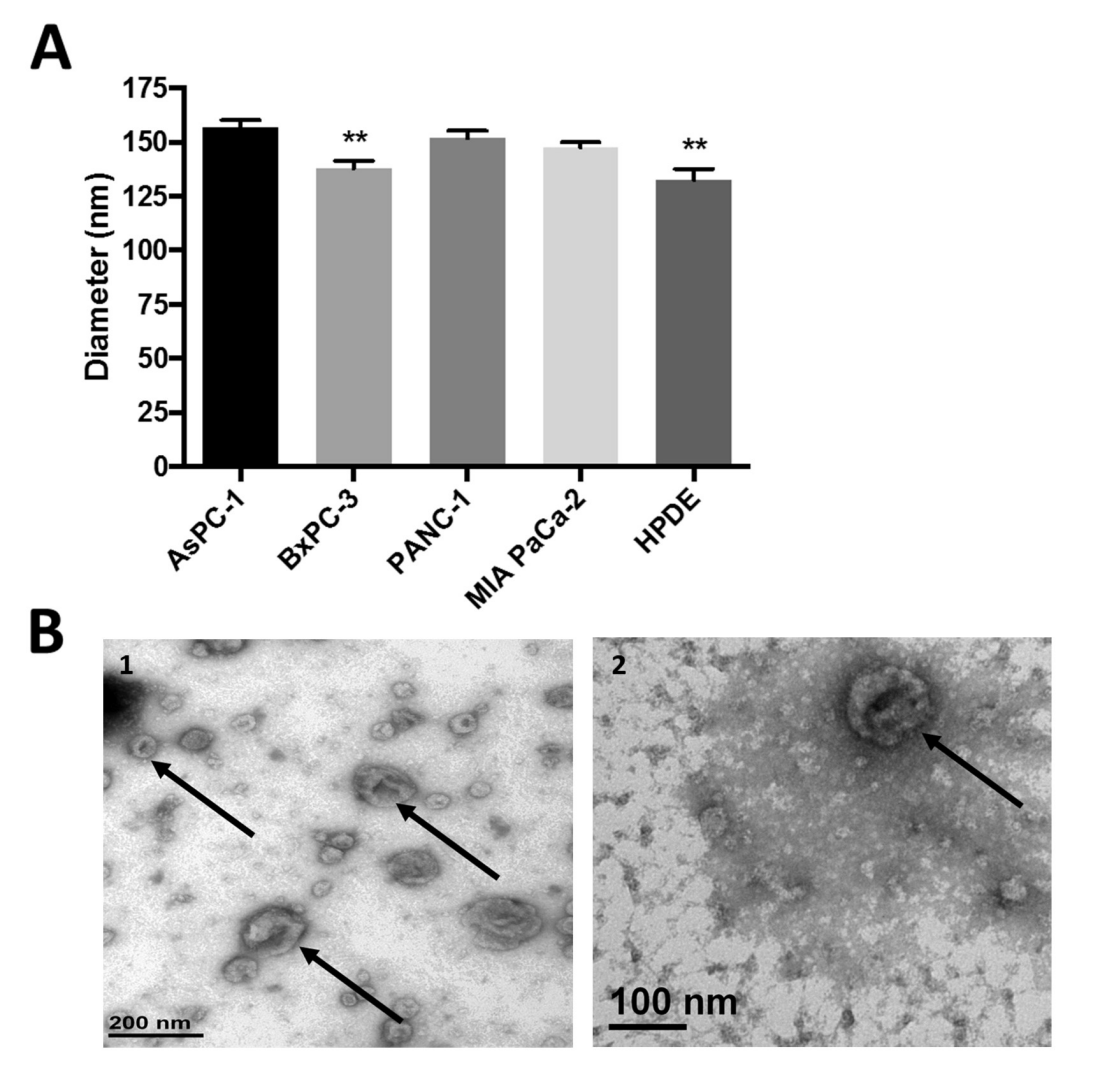
Exosomes secreted by pancreatic cancer cell lines have similar physical properties. (A) Exosomes sized by dynamic light scattering demonstrate similar average size, **p<0.005. Data from three independent experiments were combined and are represented as mean ± SEM. (B) BxPC-3 exosomes (1) and PANC-1 exosomes (2) imaged by transmission electron microscopy display size heterogeneity and characteristic cup-shaped morphology (arrows).

To physically characterize exosomes secreted by these cell lines, purified exosomes were sized using dynamic light scattering. Purified exosomes secreted by all PDAC cell lines fell within the expected size range, approximately 150 nm in diameter (Fig 1A), although exosomes from BxPC-3 and HPDE H6c7 cells were slightly smaller than exosomes from the other cell lines. When visualized by transmission electron microscopy, BxPC-3 and PANC-1 exosomes were heterogeneous yet showed the characteristic “saucer-like” morphology (Fig 1B and 1C), a finding consistent with other exosome studies which reported that populations of exosomes of varying sizes can be secreted by a single clonal cell line [20, 21].

### Analysis of exosome surface proteins

**Fig 2:**
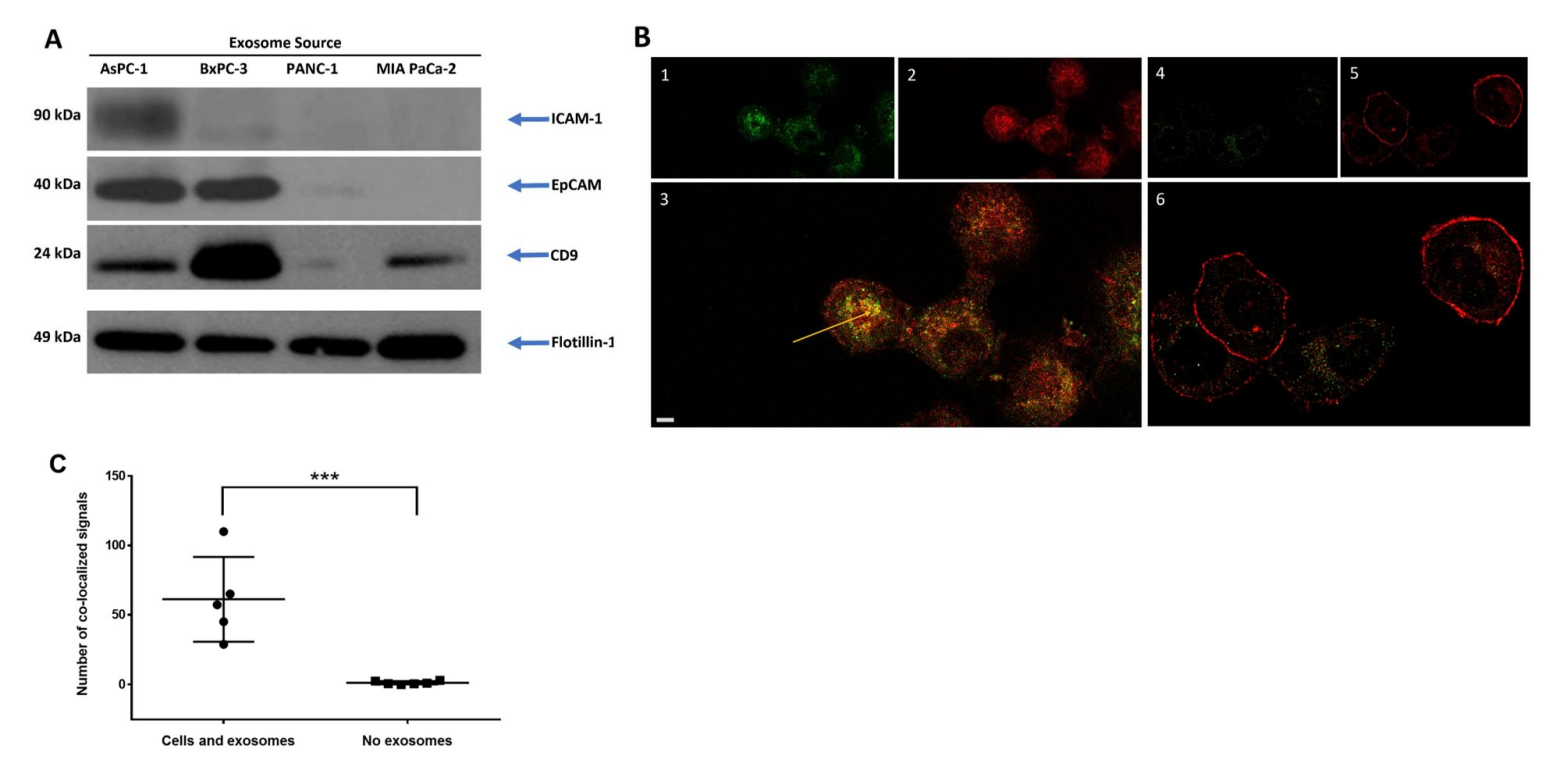
ICAM-1 and CD11c co-localize during exosome-macrophage interactions. (A) Immunoblotting analysis reveals exosomes from the pancreatic cancer cell lines AsPC-1 and BxPC-3 are enriched in the surface-exposed proteins ICAM-1 and EpCAM, while CD9 is more broadly expressed. The pan-exosomal marker flotillin-1 is used as a loading control. (B) Co-localization of exosome proteins and macrophage proteins is demonstrated by immunostaining for the exosome marker ICAM-1 (green, panel 1) or the macrophage marker CD11c (red, panel 2) after mixing AsPC-1 exosomes with THP-1-derived, non-polarized macrophages. Panel 3 is a merged image showing co-localization of ICAM-1 and CD11c staining (yellow, indicated by an arrow), and is suggestive of ICAM-1 and CD11c protein-protein interaction. THP-1-derived macrophages not mixed with exosomes (cells only) show little ICAM-1 staining (green, panel 4), suggesting exosomes were the main source of the ICAM-1 signal. In the absence of exosomes, more CD11c is associated with the THP-1 cell surface (red, panel 5), and merged images of THP-1 only staining show few areas of signal co-localization (panel 6, yellow). Scale bar = 3.0 μm (C) Quantitation of ICAM-1:CD11c co-localized signal in THP-1 cells mixed with AsPC-1 exosomes or in THP-1 cells alone. ***p<0.005.

Exosomes from all cell lines contained the pan-exosomal markers flotillin-1, a lipid-raft protein involved in exosome biogenesis, and CD9, although total CD9 protein levels differed between exosomes from different cell lines with BxPC-3 exosomes particularly enriched in CD9 (Fig 2A). Immunoblot analysis of exosomal proteins known to facilitate cell-cell or cell-matrix interactions showed that exosomes from both AsPC-1 and BxPC-3 cell lines contained high levels of EpCAM (epithelial cell adhesion molecule), and AsPC-1 exosomes showed the highest amounts of ICAM-1 (Intercellular adhesion molecule-1), which is known to interact with CD11c. CD11c is up-regulated on differentiated THP-1 macrophages [22], as well as on CD8^+^ dendritic cells and activated T cells [23]. Mutated Kras induces the expression of ICAM-1, which serves as a chemoattractant for macrophages and contributes to the formation of pancreatic intraepithelial neoplasms (PanINs), the most common precursor lesions for pancreatic ductal adenocarcinoma [24].

We hypothesized that exosomal ICAM-1 could play a role in AsPC-1 exosome interactions with CD11c^+^ macrophages. To determine if these two proteins co-localized in a mixture of AsPC-1 exosomes and THP-1-derived macrophages, the proteins were visualized using the sub-diffraction imaging technique STimulated Emission Depletion (STED) microscopy. In the exosome/macrophage mixtures, most of the anti-ICAM-1 antibody signal co-localized with the anti-CD11c antibody signal and was found internally (Fig 2B). When only THP-1-derived macrophages (no exosomes) were visualized, little ICAM-1 signal was observed, suggesting that the ICAM-1 was mainly associated with the AsPC-1 exosomes and not with the THP-1 macrophages, and the CD11c signal remained at the cell surface. Quantitation of signal co-localization showed that significantly more ICAM-1 and CD11c co-localize in THP-1 cell/exosome mixtures than in THP-1 cells alone (Fig 2C). Unlike AsPC-1 exosome/macrophage mixtures, mixtures of macrophages with PANC-1 exosomes show little co-localization of the ICAM-1 signal with the CD11c signal (S1 Fig). In addition, PANC1 exosome and macrophage mixtures demonstrated little change in localization of the CD11c signal, much of which remained at the cell surface. This suggests that the PANC-1 exosomes were less well internalized by macrophages than were AsPC-1 exosomes.

Taken together, these data show that AsPC-1 exosomes contain elevated levels of cell surface adhesion proteins ICAM-1 and EpCAM and suggest that the ICAM-1/CD11c interaction contributes to AsPC-1 exosome docking with macrophages and internalization.

### Exosomal arachidonic acid contributes to the efficiency of exosome fusion with THP-1-derived macrophages

In addition to factors that bring exosomes and target cells into close proximity, such as docking proteins, fusion between exosomes and the plasma membranes can be influenced by membrane lipids [25]. A number of fusogenic lipids have been identified, particularly arachidonic acid (AA) [26, 27]. AA is required for endocytic vesicle fusion as well as annexin II-mediated exocytosis [28]. Mast cell exosomes are enriched in AA and AA-derivatives such as prostaglandin E2 (PGE_2_) and 15-deoxy- 12, 14 –prostaglandin J2 (15-d PGJ_2)_ [29]. We therefore examined whether AA plays a role in pancreatic cancer cell exosome fusion with macrophages.

**Fig 3:**
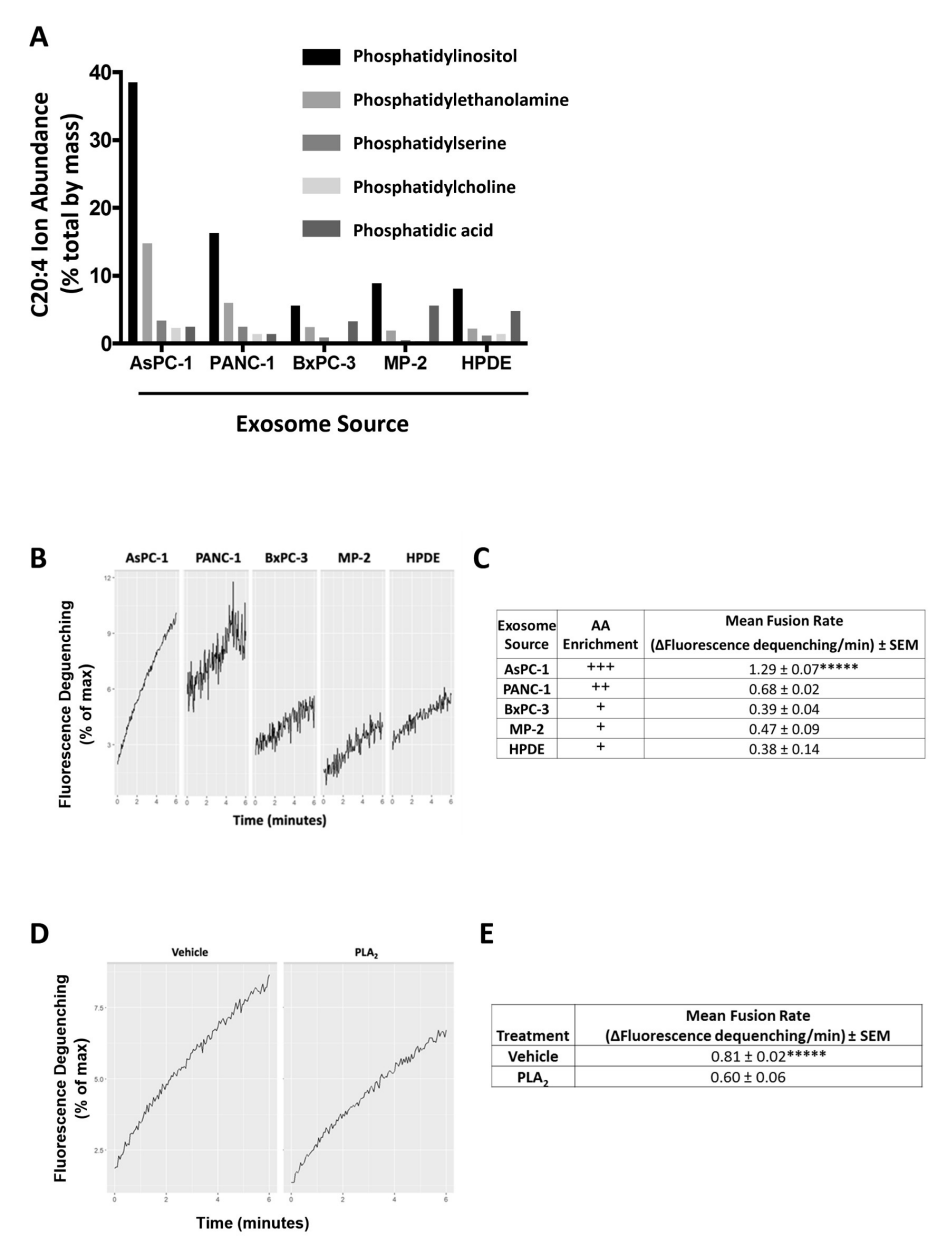
AsPC-1 exosomes are enriched in arachidonic acid and exosomal arachidonic acid contributes to the efficiency of AsPC-1 exosome-macrophage fusion. (A) Compared to exosomes from other PDAC cell lines and from HPDE cells, AsPC-1 exosomes are enriched in arachidonic acid (C20:4) species. Comparison of the percent of arachidonic acid in each glycerophospholipid class showing that the percentage of AA is highest in the PI and PE fractions. Phosphatidylcholine = PC, phosphatidylserine = PS, phosphatidylethanolamine = PE, phosphatidic acid = PA, phosphatidylinositol = PI. (B) Representative fusion curves of exosomes with non-polarized THP-1 macrophages. (C) Compared with exosomes from other pancreatic cell lines, AsPC-1 exosomes fuse at the highest rate with THP-1-derived macrophages. Relative arachidonic acid (AA) levels shown with plus signs. (D, E) Pre-treatment of AsPC-1 exosomes with phospholipase A_2_ (PLA_2_), which removes AA from phospholipids, decreased the exosome fusion rate. Data from three independent experiments are represented as mean ± SEM. *****p<0.0005 by ANOVA.

We performed a single, pilot analysis of exosomal lipid/fatty acid composition by harvesting secreted exosomes from the various cell lines. The most notable difference between exosomes from different PDAC cell lines was the much higher percentage of phospholipid-esterified AA in exosomes isolated from the ascites-derived AsPC-1 cell line (Fig 3A). Composition analysis found that the exosomal AA was found mainly in the phosphatidylinositol (PI) and phosphatidylethanolamine (PE) glycerophospholipid fractions (Fig 3A).

Functionally, PDAC exosomes fused slowly with undifferentiated THP-1 monocytes but fused more readily with activated but non-polarized (M0) THP-1 macrophages generated by PMA treatment. AsPC-1 exosomes exhibited the highest rate of fusion with THP-1 macrophages (Fig 3B and 3C). Within the first 6 minutes after mixing with macrophages, AsPC-1 exosomes were significantly more fusogenic than exosomes from PANC-1, MIA PaCa-2, BxPC-3, or HPDE cells, which fused with macrophages at similar rates (Fig 3E). No spontaneous dequenching was noted after the addition of unlabeled exosomes to R18-labeled exosomes, indicating that exosomes themselves were stable and non-fusogenic in the absence of target cells.

AsPC-1 exosomes were then treated with recombinant human phospholipase A_2_ (PLA_2_), which removes AA from the *sn*-2 position of phospholipids in the exosome membrane. PLA_2_ treatment resulted in a significant decrease in the AsPC-1 exosome fusion rate (Fig 3D and 3E). Although fusion was not completely eliminated, the fusion rate of PLA_2_-treated AsPC-1 exosomes was comparable to PANC-1 exosomes, suggesting that AA was not the only factor contributing to exosome fusion with macrophages. However, this demonstrated that AA is one determinant of the overall fusogenic potential of AsPC-1 exosomes.

### AsPC-1 exosomes polarize THP-1-derived macrophages towards an immunosuppressive M2 phenotype

**Fig 4:**
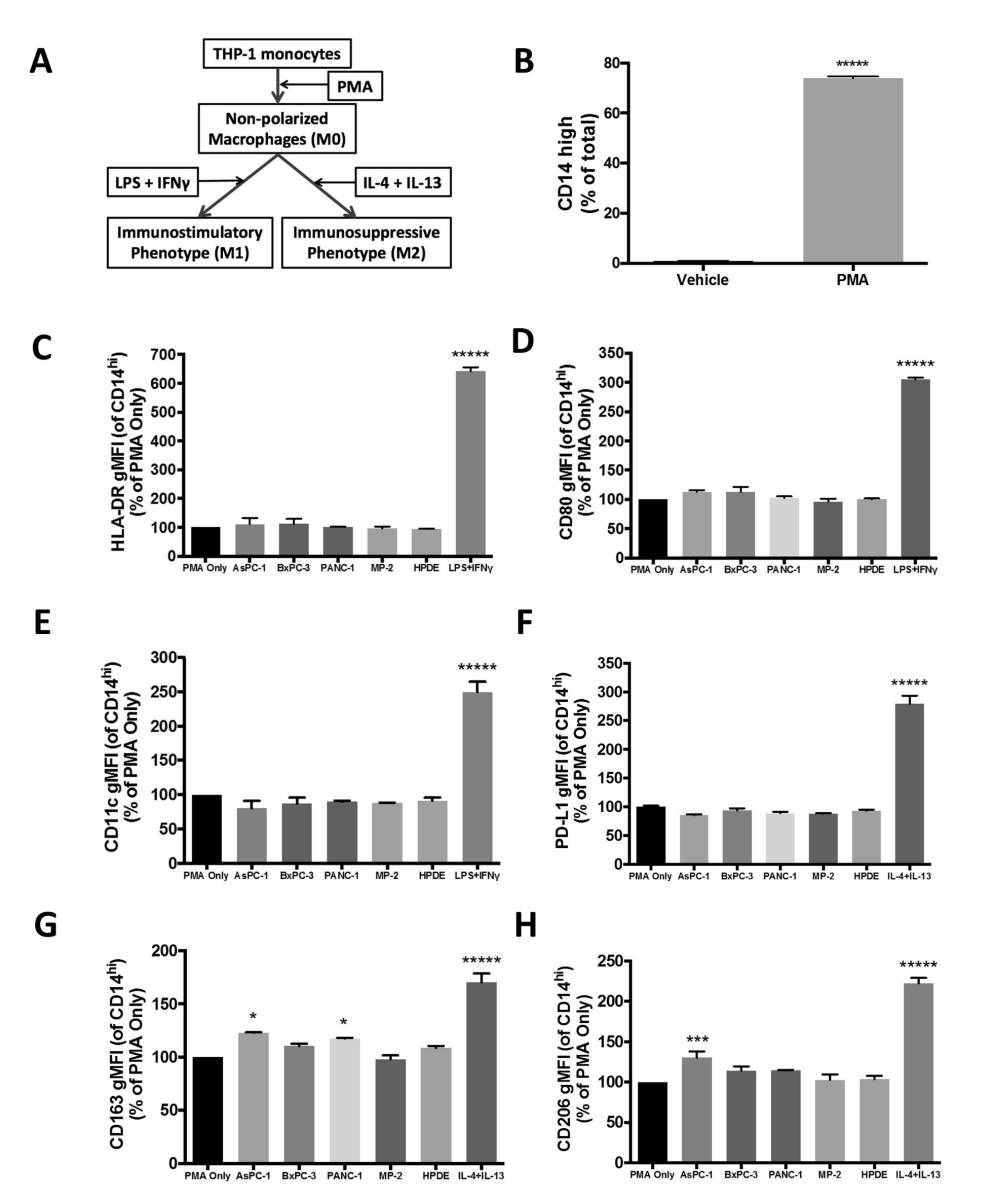
AsPC-1 exosomes polarize THP-1-derived macrophages towards an immunosuppressive M2 phenotype. (A) Workflow of *in vitro* THP-1 monocyte differentiation and positive controls for macrophage polarization. LPS + IFNɣ are M1 macrophage polarization controls, while IL-4 + IL-13 are controls for M2 macrophage polarization. (B) PMA-induced differentiation of THP-1 monocytes into non-polarized macrophages significantly increased CD14 protein levels; data from three independent experiments are represented as mean ± SEM, ****p<0.0005 by unpaired two-tailed t-test. Non-polarized CD14^hi^ macrophages treated with AsPC-1 exosomes show no change in markers of M1 polarization, HLA-DR, CD80, or CD11c (C, D, E), or in the level of the immunoregulatory protein PD-L1 (F). In contrast, CD14^hi^ macrophages treated with AsPC-1 exosomes show significantly increased levels of the M2 markers CD163 (G) and CD206 (H), and Panc-1 exosomes also increased CD163 expression. Data from at least two independent experiments are represented as mean ± SEM. *****p<0.0005, ***p<0.005, *p<0.05.

The effects of PDAC exosomes on the expression of macrophage cell surface markers were analyzed by flow cytometry (Fig 4A). We hypothesized that exosomes from PDAC cell lines would cause PMA-differentiated M0 macrophages that express CD14 (Fig 4B) to adopt an immunosuppressive phenotype that favors tumor cell invasion and metastatic spread. Treatment of THP-1 monocytes with PDAC exosomes *in vitro* did not cause the monocytes to display morphological evidence of having undergone differentiation into macrophages (i.e. adhesion to culture dish). This suggests that exosomes derived from these PDAC cell lines do not differentiate monocytes into macrophages. We then treated differentiated (PMA-treated) but non-polarized THP-1-derived M0 macrophages with exosomes and analyzed changes in phenotypic markers of M1 or M2 macrophage polarization. Treatment of THP-1-derived macrophages with PDAC exosomes did not significantly change levels of HLA-DR, CD80, or CD11c (Fig 4C–4E), all of which are associated with classically activated, immunostimulatory M1 macrophages. Although TAMs are known to express the immunoregulatory protein PD-L1, which can modify T-cell function [30], the level of macrophage PD-L1 expression was not increased after exosome addition (Fig 4F). However, treatment of THP-1 macrophages with AsPC-1 exosomes did result in significant increases in CD163 and CD206 (Fig 4G and 4H), markers associated with alternatively-activated, immunosuppressive M2 macrophages (CD14^hi^ CD163^hi^ CD206^hi^) [31]. Exosomes from other PDAC cell lines or HPDE cells did not significantly change M1 or M2 markers with the exception of PANC-1 exosomes, which also increased CD163 expression on THP-1 macrophages (Fig 4G) but did not increase CD206 levels. The observation that AsPC-1 exosomes polarized macrophages towards an M2 phenotype suggests that exosomes from this ascites-derived cell line promote pro-tumoral macrophage functions.

### AsPC-1 exosomes enhance secretion of PGE_2_, VEGF, MCP-1, IL-6, IL-1β, MMP-9, and TNFα

**Fig 5:**
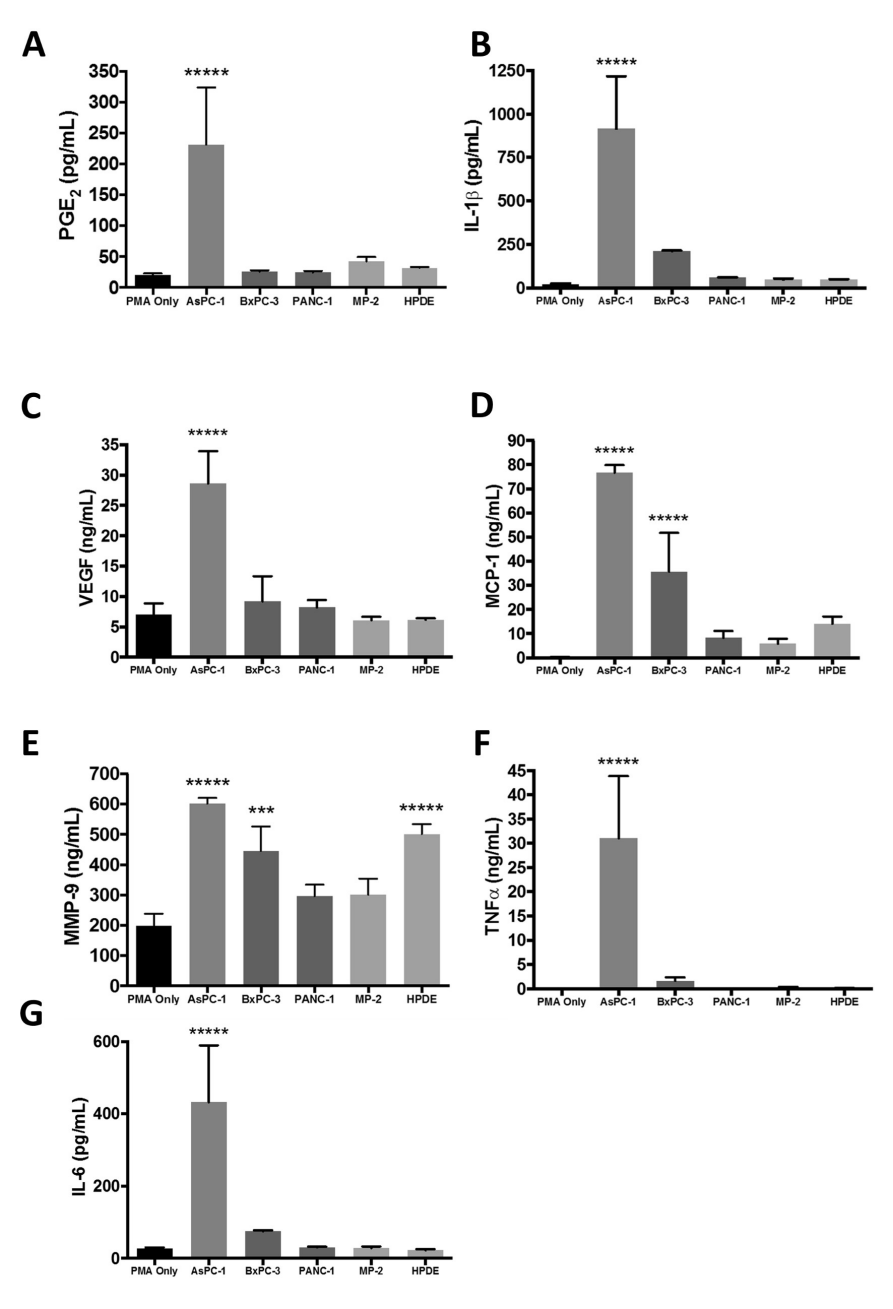
THP-1-derived macrophages treated with AsPC-1 exosomes secrete bioactive molecules associated with tumor progression, angiogenesis, invasion, and metastasis. THP-1 macrophages treated with AsPC-1 exosomes secrete elevated amounts of prostaglandin E2 (PGE_2_) (A), angiogenic factor VEGF (C), cytokines and chemokines MCP-1, IL-6, IL-1β, and TNFα (B, D, F, G) and the metalloprotease MMP-9 (E). Exosomes from BxPC-3 also stimulated macrophage secretion of MCP-1, and exosomes from both BxPC-3 and HPDE cells significantly enhanced the secretion of MMP-9. Data from at least three independent experiments are represented as mean ± SEM. *****p<0.0005, ***p<0.005, *p<0.05.

Both pancreatic tumor cells and TAMs secrete cytokines, chemokines, and other bioactive factors that shape the TME, enhance tumor growth, and influence local invasion and metastasis [31]. We hypothesized that delivery of arachidonic acid from cancer cells to macrophages via exosomes could cause a significant elevation in secretion of PGE_2_, a downstream product of AA metabolism, by macrophages. Treatment of non-polarized THP-1-derived macrophages with AsPC-1 exosomes enhanced the secretion of PGE_2_, (Fig 5A), as well as enhancing secretion of other cytokines, chemokines, and bioactive factors including IL-1β, VEGF, MCP-1, MMP-9, TNFα and IL-6 (Fig 5B–5G). Exosomes from the BxPC-3 cell line also stimulated macrophage secretion of MCP-1, suggesting that exosomes from this cell line also prompts the recruitment of additional monocytes to the tumor (Fig 5D). The effects of exosomes on secretion of MMP-9 by THP-1 cells was the most variable among cell lines. Treatment with exosomes from AsPC-1, BxPC-3 and the normal pancreatic epithelial cell line HPDE all increased the production of MMP-9, which has been linked to local invasion, while exosomes from PANC-1 and MIA PaCa-2 did not (Fig 5E). This suggests that exosomal factors other than AA and PGE_2_ influence MMP-9 secretion by macrophages. Together, these results indicate that exosomes secreted by pancreatic cancer cell lines, especially the ascites-derived, metastatic AsPC-1 cell line, can alter macrophage phenotype and enhance secretion of bioactive factors that play a role in pancreatic tumor growth and dissemination.

## Discussion

Pancreatic tumors are enriched in infiltrating immune cells, including tumor-associated macrophages (TAMs), which influence tumor cell migration, invasion, and metastasis. TAMs facilitate metastasis by synthesizing and secreting enzymes and soluble factors that contribute to cancer cell migration through ECM remodeling. Although crosstalk between PDAC tumor cells and other cells composing the TME is known to occur through soluble mediators, such as cytokines, chemokines and growth factors, the role of exosomes in cell-cell communication within the local TME is less well understood.

**Fig 6:**
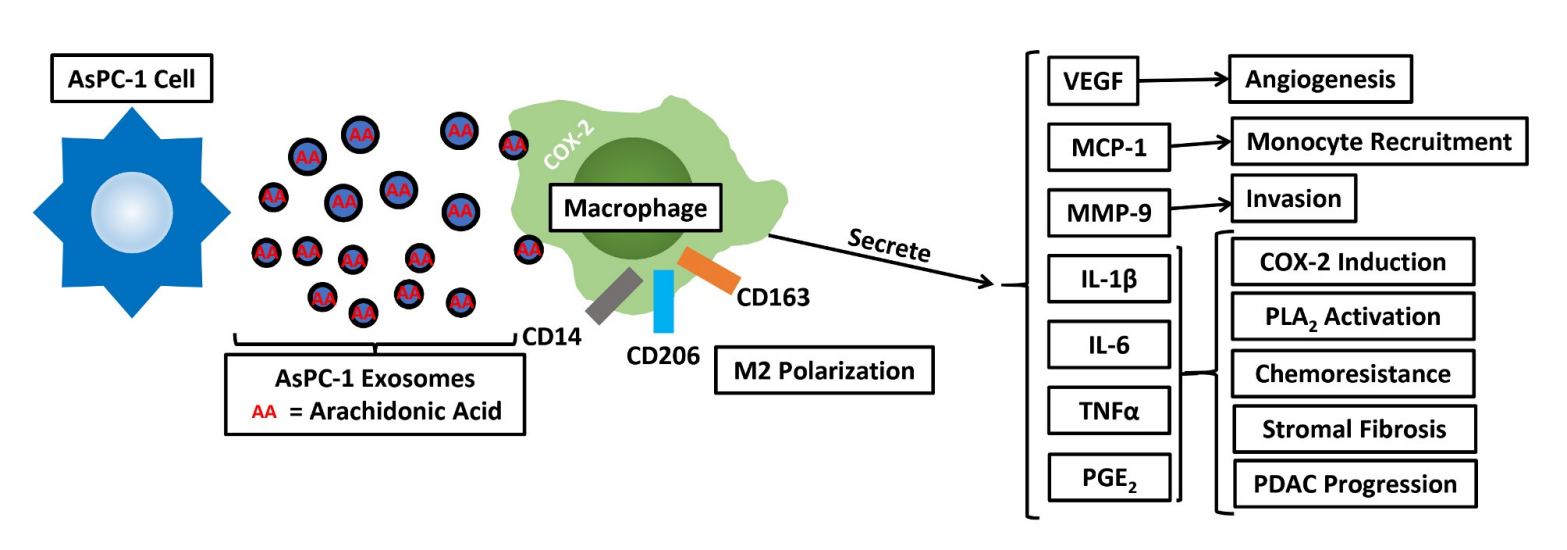
Proposed model for the immunomodulatory and pro-tumoral effects of AsPC-1 exosomes. While lipids form the basis of exosome membrane structure, exosomes may also shuttle bioactive lipid mediators, such as AA, between tumor cells and macrophages to promote a local, pro-tumor microenvironment.

This study compared the effects of exosomes from various pancreatic cancer cells and from a normal pancreatic epithelial cell line on macrophage phenotype and function. The most consistent result is that exosomes from an ascites-derived, metastatic pancreatic cancer cell line, AsPC-1, differ from exosomes derived from other less metastatic PDAC cancer cell lines derived from primary tumors and from a normal pancreatic epithelial cell line. In fact, exosomes from the normal pancreatic cell line HPDE H6c7 were surprisingly similar in many respects to exosomes from primary pancreatic tumor cell lines. Clinically, ascites are present in approximately one-third of PDAC patients and the presence of ascites is associated with peritoneal spread, significantly worse prognosis and shortened median survival [32]. The presence of tumor cells in ascites fluid is well documented, and has been used to individualize patient treatment [33]. In contrast to exosomes from other PDAC cell lines and from HPDE cells, AsPC-1 exosomes (i) are enriched in surface-exposed proteins involved in cell-cell interactions, (ii) fuse with macrophages at a higher rate, due in part to arachidonic acid, and (iii) have pro-tumorigenic phenotypic and functional effects on macrophages (Fig 6).

AsPC-1 exosomes are enriched in ICAM-1, a protein that mediates cell-cell interactions and which has been shown to direct dendritic cell exosome fusion with naïve T-cells [34]. CD11c protein levels are undetectable in undifferentiated THP-1 monocytes but are significantly elevated 24 hours after monocytes are differentiated into non-polarized macrophages with PMA [22]. While AsPC-1 exosomes fused at a low rate with undifferentiated THP-1 monocytes, the rate of fusion of AsPC-1 exosomes with PMA-differentiated THP-1 macrophages was significantly increased. Co-localization of AsPC-1 exosomal ICAM-1 with macrophage CD11c suggests that uptake of AsPC-1 exosomes occurs via protein-mediated exosome-docking at the macrophage cell surface, and ICAM-1 could also promote cancer cell exosome uptake by other CD11c^+^ immune cells in the TME or at metastatic sites [35]. However, since exosomes from other PDAC cell lines express ICAM-1 at lower or undetectable levels can still fuse with THP-1 cells, other protein-protein interactions must play a role in exosome-target cell recognition.

The AA (20:4) component of phospholipids from AsPC-1 exosomes contributes to the increased fusogenicity of these exosomes with THP-1 derived macrophages. Others have shown that AA-containing phosphatidylinositol, PI(18:0/20:4), is enriched at the cancer cell/stromal interface in colorectal cancer patient tumors [36], and levels of phosphatidylserine 18:0/20:4, phosphatidylinositol 18:0/20:4, and phosphatidylcholine 18:0/20:4 are markedly higher in metastatic MDA-MB-231 breast cancer cells than in MCF-7 cells [37]. While both proteins and lipids contribute to the overall fusogenicity of exosomes with cancer cells [38], this study demonstrates that AA in AsPC-1 exosomes is one determinant of their fusogenicity with target macrophages *in vitro*. Compared to exosomes from other PDAC cell lines, AsPC-1 exosomes were found to fuse at the highest rate with THP-1-derived macrophages, and the fusogenicity of AsPC-1 exosomes decreased after AA was removed by PLA_2_ pre-treatment. Once incorporated into the target cell membrane, AA can be converted into PGE_2_ through the activity of PLA_2_, cyclooxygenase-2 (COX-2) and microsomal prostaglandin E2 synthase 1 (mPGES1). Recent evidence suggests that PDAC M2 macrophages expressed increased levels of mPGES-1 and 5-LOX, and pharmacological inhibition of these enzymes blocked tumor progression *in vivo* [39]. Interestingly, AsPC-1 cells themselves secrete negligible amounts of PGE_2_, yet can stimulate PGE_2_ secretion when co-cultured with PBMCs [40]. We propose that AsPC-1 cells are delivering bioactive, immunomodulatory lipids, including arachidonic acid, to macrophages through exosomes. Treatment of macrophages with AsPC-1 exosomes triggered a significant increase in PGE_2_ secretion, whereas treatment of these cells with exosomes not as highly enriched in AA did not cause as significant an increase in PGE_2_ secretion. While the enhanced secretion of PGE_2_ could be responsible for the pro-tumorigenic effects AsPC-1 exosomes have on macrophages, exosomal AA may also promote cross-talk between cancer cells and the TME through the 5-lipoxygenase pathway [41].

The COX-2/mPGES1/PGE_2_ pathway is up-regulated in several cancer types, including pancreatic tumors [42]. PGE_2_ is significantly increased in PDAC patient blood and urine samples [43, 44], and higher COX-2 expression is independently correlated with a poor prognosis [45]. Within the TME, PGE_2_ signaling through the EP4 receptor activates human pancreatic stellate cells leading to extensive tumor fibrosis [46]. Activation of the PGE_2_/EP4 signaling pathway on endothelial cells also promotes both angiogenesis [47] and lymphangiogenesis [48], while stromal macrophage over-expression of COX-2 increased colonic tumor progression in Apc ^Min/+^ mice [49]. PGE_2_ enhances secretion of CXCL1, IL-6, and granulocyte colony-stimulating factor (G-CSF) by myeloid cells and can trigger differentiation and immunosuppressive activation of myeloid-derived suppressor cells (MDSCs) [50–52]. Finally, PGE_2_ stimulates and recruits pro-tumor regulatory T cells to the tumor [53], and suppresses the anti-tumor cytotoxic activity of CD8+ T cells [54]. Although a recent report demonstrated that PGE_2_-enhanced the expression of PD-L1 on murine F4/80^+^ macrophages and Ly-6C^+^ myeloid-derived suppressor cells [55], we did not observe a change in PD-L1 expression on THP-1 macrophages treated with PDAC exosomes. However, others have recently reported that PANC-1 exosomes can reprogram murine J771.A1 macrophages or human monocytes to an M2-like (CD14^+^HLA-DR^lo^, increased Arg1) phenotype [56, 57]. Thus, the bioactive lipids from tumor cell exosomes could trigger pro-inflammatory pathways in several TME cell types.

In addition to enhancing M2-polarization and the secretion of PGE_2_ by THP-1 macrophages, AsPC-1 exosomes increased the secretion of several other bioactive growth factors, chemokines and cytokines including VEGF, MCP-1, IL-6, IL-1β, MMP-9, and TNFα, all of which have been shown to contribute to pancreatic tumor growth and progression (Fig 6). MCP-1 (CCL2) enhances recruitment and infiltration of monocytes into PDAC tumors and promotes M2 macrophage polarization. Tumor exosomes have been shown to activate MDSCs by inducing autocrine IL-6 production, which is further linked to STAT3 activation and response to chemotherapy [58, 59]. IL-1β induces COX2 expression in PDAC cells, suggesting the potential for a positive feedback loop to further increase PGE_2_ secretion within the TME, and increases tumor fibrosis [60, 61]. Interestingly, a recent systematic analysis of changes in gene expression across many cancer types identified AA metabolism as a major pathway dysregulated in cancer cells [62].

This study demonstrates that exosomes secreted by AsPC-1 pancreatic cancer cells, which contain increased amounts of the fusogenic and bioactive fatty acid AA, contribute to enhanced secretion of PGE_2_, other cytokines, and pro-angiogenic and metastatic factors by macrophages. While other studies of pancreatic cancer exosome function have focused on the role of exosomes in priming the pre-metastatic niche in distant organs [6], this study reveals the immunosuppressive and pro-metastatic effects that pancreatic cancer exosomes have within the local TME and suggests that these exosomes are a key contributor to disease progression.

## Supporting information

S1 Fig ICAM-1 and CD11c in exosome-target cell interactions. Mixtures of PANC-1 exosomes with THP-1-derived, non-polarized macrophages show little co-localization of ICAM-1 (green, panel 1) with the macrophage marker CD11c (red, panel 2). A merged image (panel 3) showing few areas of co-localized ICAM-1 and CD11c staining (yellow) and little relocalization of CD11c from the cell surface to the cytosol. Scale bar = 3.4 μm.
(TIFF)
