# Supplementary figures and images for: Tumor-promoting effects of pancreatic cancer cell exosomes on THP-1-derived macrophages
Source: PLoS One. 2018 Nov 1;13(11):e0206759. doi: 10.1371/journal.pone.0206759 (PMC6211741; doi:10.1371/journal.pone.0206759)

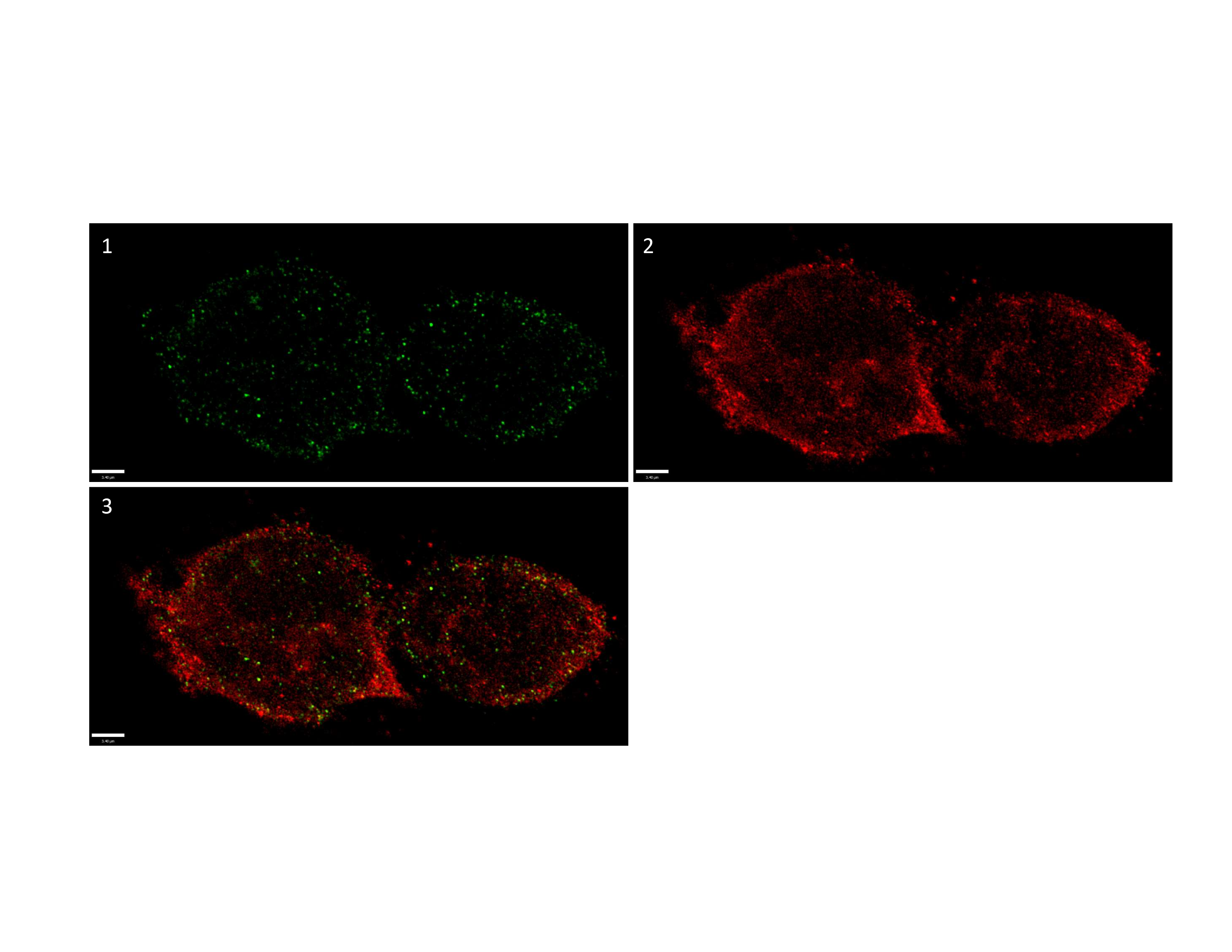

Supplement: S1 Fig — Mixtures of PANC-1 exosomes with THP-1-derived, non-polarized macrophages show little co-localization of ICAM-1 (green, panel 1) with the macrophage marker CD11c (red, panel 2). A merged image (panel 3) showing few areas of co-localized ICAM-1 and CD11c staining (yellow) and little relocalization of CD11c from the cell surface to the cytosol. Scale bar = 3.4 μm. (TIFF) [file pone.0206759.s001.tiff]
